# Assessing the Burden of Dengue during the COVID-19 Pandemic in Mexico

**DOI:** 10.3390/tropicalmed8040232

**Published:** 2023-04-19

**Authors:** Agustin Lugo-Radillo, Oliver Mendoza-Cano, Xóchitl Trujillo, Miguel Huerta, Mónica Ríos-Silva, José Guzmán-Esquivel, Verónica Benites-Godínez, Jaime Alberto Bricio-Barrios, Eder Fernando Ríos-Bracamontes, Martha I. Cárdenas-Rojas, Yolitzy Cárdenas, Efrén Murillo-Zamora

**Affiliations:** 1 CONACyT—Facultad de Medicina y Cirugía, Universidad Autónoma Benito Juárez de Oaxaca, Ex Hacienda Aguilera S/N, Carr. a San Felipe del Agua, Oaxaca 68020, Mexico; 2 Facultad de Ingeniería Civil, Universidad de Colima, km. 9 Carretera Colima-Coquimatlán, Coquimatlán 28400, Mexico; 3 Centro Universitario de Investigaciones Biomédicas, Universidad de Colima, Av. 25 de Julio 965, Col. Villas San Sebastián, Colima 28045, Mexico; 4 Centro Universitario de Investigaciones Biomédicas, CONACyT—Universidad de Colima, Av. 25 de Julio 965, Col. Villas San Sebastián, Colima 28045, Mexico; 5 Unidad de Investigación en Epidemiología Clínica, Instituto Mexicano del Seguro Social, Av. Lapislázuli 250, Col. El Haya, Villa de Álvarez 28984, Mexico; 6 Coordinación de Educación en Salud, Instituto Mexicano del Seguro Social, Calzada del Ejercito Nacional 14, Col. Fray Junípero Serra, Tepic 63160, Mexico; 7 Unidad Académica de Medicina, Universidad Autónoma de Nayarit, Ciudad de la Cultura “Amado Nervo”, Tepic 63155, Mexico; 8 Facultad de Medicina, Universidad de Colima, Av. Universidad 333, Col. Las Víboras, Colima 28040, Mexico; 9 Departamento de Medicina Interna, Hospital General de Zona No. 1, Instituto Mexicano del Seguro Social, Av. Lapislázuli 250, Col. El Haya, Villa de Álvarez 28984, Mexico

**Keywords:** dengue, global burden of disease, disability-adjusted life years, COVID-19

## Abstract

The transmission of the dengue virus in Mexico has historically been high, and its burden during the COVID-19 pandemic is currently not well understood. Our objective was to assess the burden of dengue-related disability-adjusted life years (DALYs) between 2020 and 2022. We conducted a cross-sectional analysis of databases resulting from an epidemiological surveillance of vector-borne diseases and computed DALYs using the protocol of the Global Burden of Disease (GBD) study 2019. Our results showed that there were 218,807 incident cases of dengue during the study period, resulting in 951 deaths. The calculated DALYs (and their 95% confidence intervals) were 8121 (7897–8396), 4733 (4661–4820), and 8461 (8344–8605) in 2020, 2021, and 2022, respectively. The DALY rates (per 100,000) were 6.5 (6.3–6.6), 3.8 (3.7–3.9), and 6.7 (6.6–6.8), respectively. The rates for 2020 and 2022 were similar to the historical mean (6.4, *p* = 0.884), whereas the rate for 2021 was lower than the mean. Premature mortality (years of life lost, YLL) contributed to 91% of the total burden. Our findings suggest that dengue fever remained a significant cause of disease burden during the COVID-19 pandemic, especially in terms of premature mortality.

## 1. Introduction

Dengue fever is a viral infection transmitted by arthropods, with a high social and economic burden in most tropical and subtropical regions of the world, including Mexico [1]. The main vectors of this viral pathogen are Aedes (Ae.) aegypti and Ae. albopictus mosquitoes, which are found in twenty-eight and fourteen out of the thirty-two states of the country, respectively [2,3].

The COVID-19 pandemic has significantly impacted the diagnosis and treatment of vector-borne diseases from multiple perspectives [4]. It has disrupted health systems and diverted resources away from vector-borne disease control programs. In addition, social distancing measures and travel restrictions have limited the ability of public health officials to conduct surveillance and control measures [5]. Therefore, the pandemic has highlighted the importance of maintaining strong and resilient public health systems that can respond to multiple health threats simultaneously. Currently, the burden of dengue during this period in Mexico is unknown.

The methods of the Global Burden of Disease (GBD) study provide a comprehensive approach to quantify health loss [6]. Disability-adjusted life years (DALYs), which are synthetic estimates obtained, are highly useful to assist health decision-makers in planning, prioritizing, and allocating health resources [7]. The two components used to calculate DALYs are years lived with disability (YLD) and years of life lost (YLL). YLD measures the years of healthy life lost due to disability, while YLL measures the years of life lost due to premature death [8].

The aim of this study was to assess the disease burden of dengue fever in Mexico from 2020 to 2022, coinciding with the COVID-19 pandemic. Additionally, we compared our burden estimates from 2020 to 2022 with historical rates from 1990 to 2019.

## 2. Methods

We conducted a cross-sectional analysis of nationwide publicly available datasets (https://www.gob.mx/salud/documentos/datos-abiertos-bases-historicas-de-enfermedades-transmitidas-por-vector, accessed on 27 February 2023) made public by the General Directorate of Epidemiology of Mexico. This organization coordinates the epidemiological surveillance of vector-borne diseases and other events of interest for public health across the country [9]. The retrieved information included the number of registered incident cases of dengue fever (International Statistical Classification of Diseases and Related Health Problems, 10th revision [ICD-10], A90–A91) from 2020 to 2022, sorted by the date of symptom onset, gender, and age group (0–4, 5–14, 15–29, 30–44, 45–59, 60–69, 70–79, and 80+ years old). As of the 2022 data collection date, information from weeks 01 to 47 was available.

We also collected disease outcomes (recovery or death) from the audited databases. Patients who required hospital admission were classified as severe dengue cases.

The gender- and age-stratified populations of Mexico in 2020 (https://www.inegi.org.mx/app/tabulados/interactivos/?pxq=Poblacion_Poblacion_01_e60cd8cf-927f-4b94-823e-972457a12d4b&idrt=123&opc=t, accessed on 1 March 2023) and 2021–2022 (http://www.conapo.gob.mx/work/models/CONAPO/Datos_Abiertos/Proyecciones2018/pob_ini_proyecciones.csv, accessed on 1 March 2023) were used to compute dengue incidence and mortality rates.

We also calculated the dengue-related years of life lost (YLL), years lived with disability (YLD), and disability-adjusted life years (DALYs) during the study period following the protocol of the Global Burden of Diseases, Injuries, and Risk Factors study (GBD) 2019 [10]. The average age at death in the audited database, per gender and age group, was used to obtain the estimators of interest. We also used Life Tables of the Global Health Observatory to collect the life expectancy (gender- and age-stratified) for the Mexican population (https://apps.who.int/gho/data/view.main.61060, accessed on 1 March 2023). Finally, the disability weights were those used in the GBD 2019 (https://ghdx.healthdata.org/record/ihme-data/gbd-2019-disability-weights, accessed on 1 March 2023) for non-severe and severe dengue cases, respectively.

We used chi-squared and *t*-tests to compare proportions and means, respectively. The significance level (α) was set at 5%. As we analyzed fully de-identified and publicly available data solely for academic purposes, the requirement for an evaluation of the research protocol by an ethics committee was waived.

## 3. Results

Data from 218,807 cases of dengue fever were analyzed. The majority of cases occurred in females (53.4%) and were classified as non-severe (82.3%). The overall incidence rates (per 100,000) were 95.6, 28.7, and 45.4 in 2020, 2021, and 2022, respectively. Age-specific incidence rates are shown in Supplementary Figure S1.

The proportion of severe dengue cases was higher in 2021–2022 (21.4% and 22.1%, respectively) compared to 2020 (14.3%), and this difference was statistically significant (Pearson’s χ^2^ = 2.0 × 10^3^, *p* < 0.001). We also identified 951 deaths, with mortality rates of 5.9 (2020), 3.6 (2021), and 6.0 (2022) per million inhabitants.

**Figure 1:**
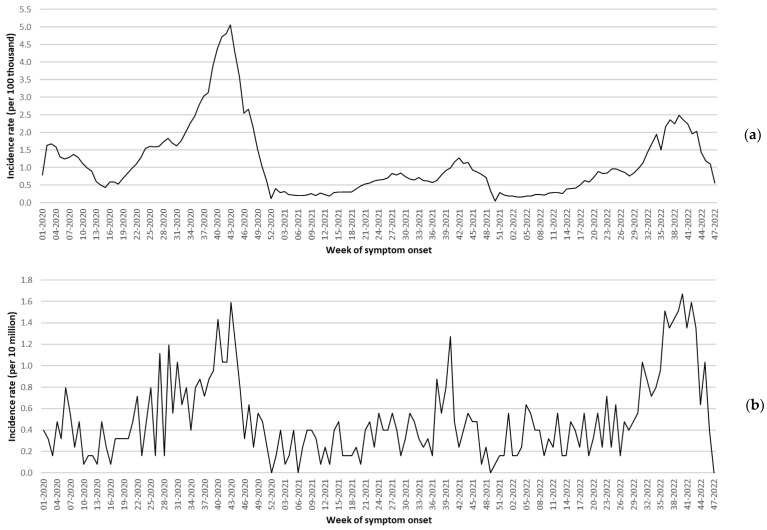
Incidence rates of non-fatal (**a**) and fatal (**b**) dengue fever cases, Mexico 2020–2022. Note: by the date of the 2022 data collection, information from weeks 48 to 52 was not available.

Figure 1a,b shows the weekly incidence rates of non-fatal (per 100,000) and fatal (per 10 million) dengue fever cases. Prior to the identification of the first COVID-19 cases in the Mexican territory (which occurred by the end of week 09-2020), a low incidence of dengue was observed. In general, higher rates of fatal cases were observed during the last trimester of each analyzed year, particularly in 2022.

Table 1 presents the disability-adjusted life years (DALYs) calculated for each year nd age group. A total of 1202 DALYs were computed from 1 January 2020 to 23 February 2020 (weeks 1 to 8), which was before the identification of the first laboratory-confirmed COVID-19 case in Mexico. These DALYs accounted for approximately 15% of the total DALYs related to dengue fever (8121 [7897–8396]) during the first year of the pandemic.

A higher count of DALYs was observed in younger individuals. For instance, in 2022, 65% of the total DALYs (5520) were computed in children and young adults aged 5 to 44 years old. Premature mortality (YLL) was the major contributor, and in any given year and age group, YLL accounted for 91% or more of the total burden.

**Figure 2:**
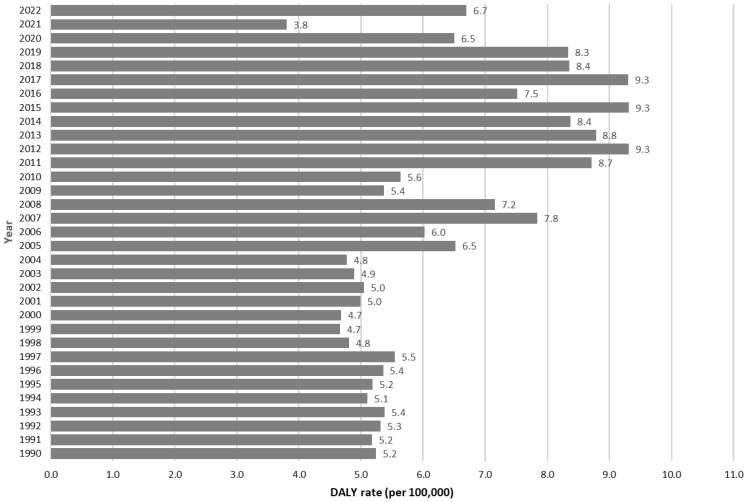
Disability-adjusted life year (DALY) rate of dengue fever, Mexico 1990–2022.

Figure 2 presents the DALY rates related to dengue fever from 1990 to 2022. The historical mean rate (1990–2019) was 6.4 per 100,000. Therefore, only the 2021 rate (3.8 per 100,000) was lower than this estimate. The rates for 2020 and 2022 (6.5 and 6.7 per 100,000, respectively) were similar to the historical mean (29 and 1 degrees of freedom (d.f.), respectively; t-value = −0.148; *p* = 0.884).

## 4. Discussion

We conducted a study to determine the burden of dengue fever in Mexico from 2020 to 2022, a period that coincided with the COVID-19 pandemic. Our findings suggest that the burden of this arthropod-borne disease decreased in the second year of the pandemic (2021) when compared with historical rates, but dengue remained a significant cause of morbidity and mortality in the population under analysis.

It is currently unclear whether the low DALY rate in 2021 resulted from low arboviral transmission or an underreporting of dengue cases [11]. We believe that a combination of both scenarios is highly plausible, at least in the context of Mexico.

Factors that may have contributed to the low dengue transmission during the COVID-19 pandemic include reduced human movement and reduced time spent in high-risk non-residential environments [12]. As shown in Figure 1a,b, the incidence of dengue fever spiked at the end of 2021 when there was high demand for health services [13]. The patterns of health service utilization in our country changed due to hospital conversions, and fear among the population of receiving medical care was also identified [14]. Therefore, it is likely that a significant number of dengue patients with mild symptoms chose to treat the disease at home rather than seek medical attention. This may also explain the 2022 profile, where a relatively low incidence of dengue fever was observed (45.4 per 100,000), but the mortality rate was high (6.0 per million). Severe dengue cases were more likely to seek healthcare and be reported.

Published evidence on dengue transmission during the COVID-19 pandemic in other populations presents heterogeneous findings. Higher rates than the historical mean were reported in Ecuador [15], Pakistan [16], Peru [17], and Singapore [18], while lower counts were observed in other countries such as Nepal [19], Brazil [20], and Colombia [21]. Interestingly, in settings such as Sri Lanka, low transmission rates were registered in school-aged children [22], and the effect of school gatherings on the dynamics of dengue epidemics in subtropical areas was previously documented [23].

We must acknowledge the potential limitations of our study. Firstly, the audited databases of vector-borne diseases do not distinguish cases according to disease severity (i.e., non-severe dengue fever versus severe dengue fever). Therefore, we used the dichotomous hospitalization variable (no/yes) as a proxy for severe disease. In these cases, higher disability weights were used when compared to those with non-severe manifestations, following the GBD 2019 study procedure.

Secondly, our analysis only considered dengue cases that were registered in the system for the epidemiological surveillance of vector-borne diseases. Therefore, the presented results might not entirely reflect the real burden of dengue during the COVID-19 pandemic. However, given the strengths of the audited system [24], the counted DALYs can be considered a lower bound of the dengue burden and are still useful from a public health perspective.

Thirdly, our study underscores the ongoing importance of dengue fever as a significant cause of disease burden during the COVID-19 pandemic, which highlights the need for robust and adaptable public health systems in our region capable of responding to multiple health threats simultaneously.

Finally, we would like to emphasize that at the time of data collection, information from 2022 was not available for 48 out of 52 weeks. As shown in Figure 1a,b, dengue incidence in Mexico has been notably high during the winter months in the northern hemisphere. Therefore, the calculated DALYs for the last analyzed year may be slightly higher compared to our current estimates.

## 5. Conclusions

Our findings suggest that dengue fever continues to pose a significant disease burden during the COVID-19 pandemic, particularly in terms of premature mortality. Although COVID-19 has been the primary focus of the global health community, our research indicates that dengue fever remains an ongoing public health challenge.

## Figures and Tables

**Table 1:** Dengue fever-related disability-adjusted life years (DALYs), Mexico 2020–2022.

Year/Age Group	DALYs (95% CI)	% YLL	DALY Rate
**2020**			
0–4	413 (404–424)	94	4.1
5–14	1333 (1293–1382)	92	6.1
15–29	2003 (1935–2087)	91	6.4
30–44	1716 (1659–1785)	91	6.4
45–59	1575 (1541–1617)	94	7.6
60–69	506 (496–517)	95	6.0
70–79	419 (415–425)	97	9.4
80+	157 (155–159)	97	7.1
All	8121 (7897–8396)	92	6.5
**2021**			
0–4	519 (515–525)	98	5.2
5–14	793 (779–810)	95	3.7
15–29	1011 (989–1037)	94	3.2
30–44	833 (817–853)	95	3.1
45–59	918 (908–931)	97	4.4
60–69	319 (316–323)	97	3.8
70–79	214 (213–216)	98	4.8
80+	124 (123–125)	98	5.6
All	4733 (4661–4820)	96	3.8
**2022**			
0–4	613 (606–621)	97	6.1
5–14	1330 (1303–1363)	94	6.1
15–29	2376 (2341–2419)	96	7.6
30–44	1814 (1790–1845)	96	6.7
45–59	1454 (1438–1473)	97	7.0
60–69	419 (414–425)	97	4.9
70–79	338 (336–342)	98	7.6
80+	118 (116–119)	97	5.3
All	8461 (8344–8605)	96	6.7

Abbreviations: CI, confidence interval; YLL, years of life lost. Notes: (1) rates per 100,000 are presented; (2) a total of 1202 DALYs were computed in weeks 01–08 of 2020, before the identification of the first laboratory-confirmed COVID-19 case in Mexican territory.

## Data Availability

Publicly available datasets were analyzed in this study. This data can be found here: https://www.gob.mx/salud/documentos/datos-abiertos-bases-historicas-de-enfermedades-transmitidas-por-vector (accessed on 27 February 2023).

## Supplementary Materials

The following supporting information can be downloaded at: https://www.mdpi.com/article/10.3390/tropicalmed8040232/s1, Figure S1 (incidence rates (per 100,000) of dengue fever per age group).
