# Supplementary material for: Assessing the Burden of Dengue during the COVID-19 Pandemic in Mexico
Source: Trop Med Infect Dis. 2023 Apr 19;8(4):232. doi: 10.3390/tropicalmed8040232 (PMC10140831; doi:10.3390/tropicalmed8040232)
Supplement: Supplementary file 1 [file tropicalmed-08-00232-s001.zip › tropicalmed-2325158-supplementary.pdf]

Burden of dengue in Mexico during the COVID-19 pandemic

Supplementary data

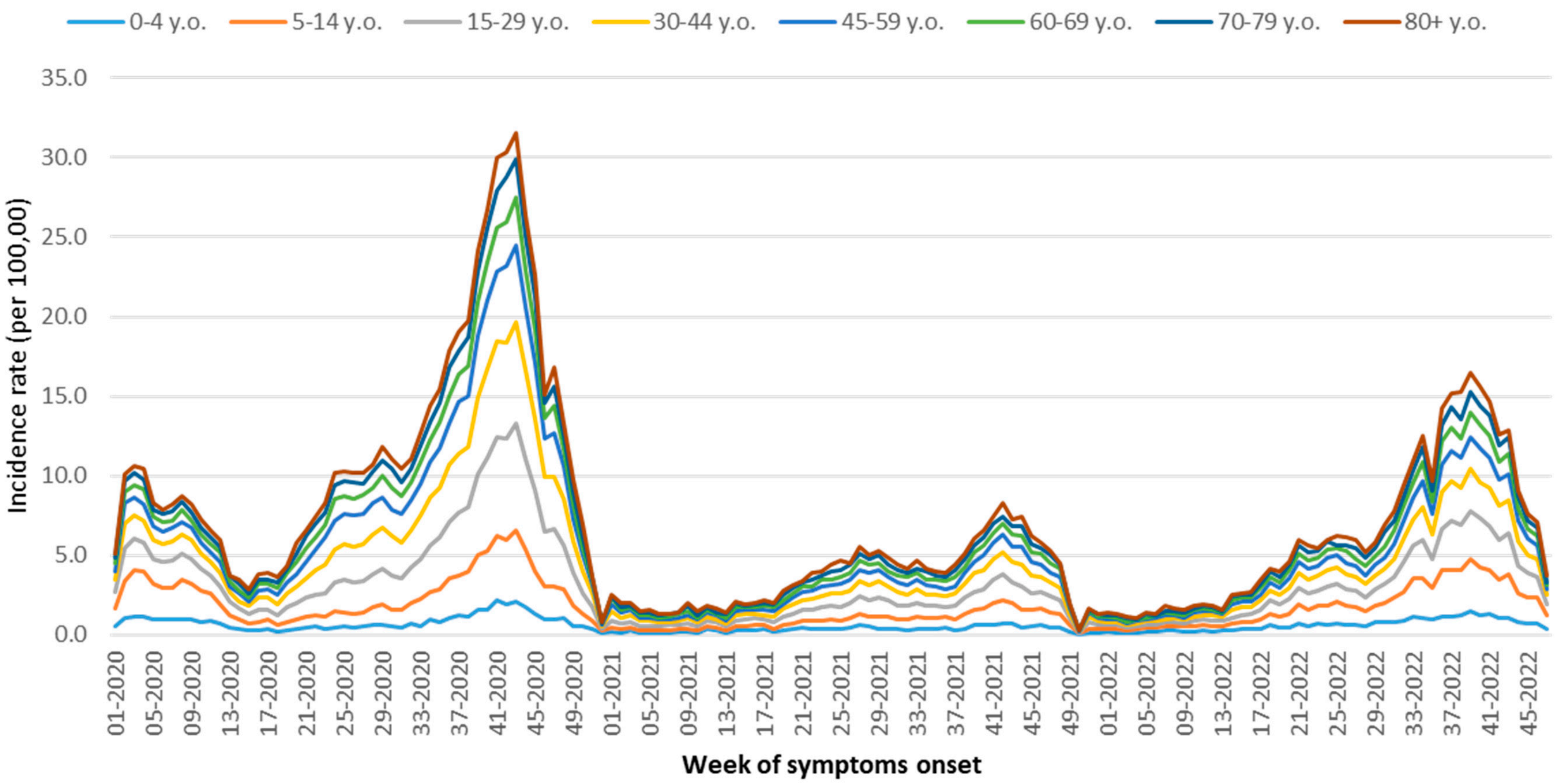

Supplementary Figure S1. Incidence rates (per 100,000) of dengue fever per age group, Mexico 2020–2022
